# The Effect of Albumin on MRP2 and BCRP in the Vesicular Transport Assay

**DOI:** 10.1371/journal.pone.0163886

**Published:** 2016-10-05

**Authors:** Feng Deng, Noora Sjöstedt, Heidi Kidron

**Affiliations:** Centre for Drug Research, Division of Pharmaceutical Biosciences, Faculty of Pharmacy, University of Helsinki, Helsinki, Finland; Russian Academy of Medical Sciences, RUSSIAN FEDERATION

## Abstract

The ABC transporters multidrug resistance associated protein 2 (MRP2) and breast cancer resistance protein (BCRP) are of interest in drug development, since they affect the pharmacokinetics of several drugs. Membrane vesicle transport assays are widely used to study interactions with these proteins. Since albumin has been found to affect the kinetics of metabolic enzymes in similar membrane preparations, we investigated whether albumin affects the kinetic parameters of efflux transport. We found that albumin increased the V_max_ of 5(6)-carboxy-2’,7’-dichlorofluorescein (CDCF) and estradiol-17-β-D-glucuronide uptake into MRP2 vesicles in the presence of 0.1% bovine serum albumin (BSA) by 2 and 1.5-fold, respectively, while BSA increased Lucifer yellow uptake by 30% in BCRP vesicles. K_m_ values increased slightly, but the change was not statistically significant. The effect of BSA on substrate uptake was dependent on the vesicle amount, while increasing BSA concentration did not significantly improve substrate uptake. These results indicate a minor effect of albumin on MRP2 and BCRP, but it should be considered if albumin is added to transporter assays for example as a solubilizer, since the effect may be substrate or transporter specific.

## Introduction

ATP-binding cassette proteins, also known as ABC proteins or ABC transporters, are a large group of membrane proteins that translocate a wide range of substrates across various biological membranes using ATP as energy [1]. ABC transporters have an important role in the disposition and elimination of endogenous compounds and the defense of the cell against xenobiotics. Due to their expression in many pharmacokinetically important tissues, some of these transporters can also affect the pharmacokinetics of drugs. Among the ABC transporters implicated in drug transport are the multidrug resistance associated protein 2 (MRP2) and the breast cancer resistance protein (BCRP) or ABCC2 and ABCG2, respectively, according to ABC nomenclature. They are involved in drug efflux in several tissues, including the liver, intestine and kidney [2–5]. MRP2 has an important role in the excretion of endogenous metabolites of estradiol and bilirubin. Dysfunction of the protein results in the accumulation of bilirubin conjugates, which is known as Dubin-Johnson syndrome [6, 7]. In addition to the physiological substrates, MRP2 is also associated with the excretion of anionic drugs and their glucuronide and glutathione conjugates (reviewed in [8]). Changes in MRP2 function or expression can cause altered exposure to drugs as has been shown for example for pravastatin [9, 10] and methotrexate [11]. Although many compounds can inhibit MRP2, so far there has been no evidence of clinical drug-drug interactions or MRP2-mediated drug induced liver toxicity. Therefore it is currently recommended that interaction of an investigational new drug with MRP2 is studied mainly if *in vivo* results indicate problems with hyperbilirubinemia [12].

BCRP is a half-transporter that was named based on its identification in a breast cancer cell line [13]. In addition to its chemotherapeutic substrates, BCRP is known to transport drugs such as sulfasalazine and rosuvastatin and is inhibited by several immunosuppressants, calcium channel blockers and HIV protease inhibitors (for a recent review see [14]). Inhibition of BCRP has been shown to increase exposure to rosuvastatin and topotecan in clinical trials [14, 15]. The Q141K genetic variant resulting in decreased expression of BCRP has been linked to altered exposure to drugs such as statins [16] as well as incidence of gout through decreased excretion of uric acid [17, 18]. Due to this influence on pharmacokinetics, BCRP is one of the transporters included in drug-drug interaction guidelines of the United States Food and Drug Administration and the European Medicines Agency [19, 20].

There are several ways of studying drug transport *in vitro* and they have been reviewed in detail by Brouwer et al. [21]. These include uptake or permeability studies using intact cells or cell monolayers overexpressing the transporter of interest, studies in sandwich cultured hepatocytes as well as uptake into inverted membrane vesicles. The methods differ in their level of complexity and the type of information they give and are therefore at best used to complement each other.

Inverted membrane vesicles with overexpressed transporters allow a way to study the interaction and kinetics of a compound with direct access of the compound to the transporter binding site [21, 22]. In the vesicular transport assay, uptake of substrates is quantified by measuring their accumulation into inside-out oriented membrane vesicles. These vesicle-based assays allow a higher throughput and convenience compared to whole cell assays since they can be prepared in large batches and cryopreserved for later use. The interaction data generated in the vesicle system can be used to predict the *in vivo* transporter-mediated disposition and possible drug-drug interactions. However, the reliability and usefulness of vesicle-based predictions remains to be clarified [21].

Similar membrane vesicle preparations are used to study the interaction of drugs with metabolizing enzymes, like UDP-glucuronosyltransferase (UGT) and Cytochrome P450. However, *in vitro* based extrapolations of clearance using these vesicles tend to underpredict *in vivo* clearance [23]. One factor seen to increase the activity of UGTs and CYP 450 metabolic enzymes *in vitro* and thus alleviate underprediction has been the use of human or bovine serum albumin (BSA) in metabolic assays. For example Kilford et al. [24] showed improved predictions of intrinsic clearance for 8 out of 10 drugs metabolized by CYPs and/or UGTs mainly through CYP2C9 and UGT2B7 effects when 2% BSA was included in assays [24]. Beneficial effects of albumin on clearance predictions have also been demonstrated for compounds such as phenytoin [25–27] and lamotrigine [28].

Due to the similarity of the preparations used to study the metabolic enzymes and the transporters that may affect drug pharmacokinetics, we investigated whether BSA has an effect on the observed ATP-dependent transport of the ABC transporters MRP2 and BCRP in the vesicular transport assay and if the effect could potentially be mediated by fatty acids as has been proposed for the metabolic enzymes.

## Materials and Methods

### Materials

Human BCRP cDNA, pcDNA3-BCRP (R482T mutant)[13] and human MRP2 cDNA (pGEM3-MRP2) corresponding to NCBI Genbank accession number entry U49248, were kind gifts from Dr Douglas Ross (University of Maryland Greenbaum Cancer Center) and Dr Piet Borst (The Netherlands Cancer institute, Netherlands), respectively. Essentially fatty acid free bovine serum album (BSA) was purchased from Sigma-Aldrich (St. Louis, MO). The water soluble cholesterol/RAMEB (randomly methylated beta-cyclodextrin) complex was obtained from Cyclolab Ltd (Hungary).

### Vesicle preparations

Recombinant baculovirus for expressing MRP2, BCRP and β-galactosidase (for control membranes) were created according to the Bac-to-bac® Baculovirus Expression system protocol (Invitrogen Life Technologies, Waltham, MA). The MRP2 and β-galactosidase pFastBacI plasmids were constructed as described in. [29]. The BCRP-pFastBacI plasmid was generated by subcloning either BCRP cDNA from pcDNA3-BCRP into the BamHI/XhoI site. The BCRP cDNA was converted to wildtype BCRP (corresponding to Uniprot entry Q9UNQ0) with QuikChange® Lightning Site-Directed Mutagenesis Kit and primers 5´-ctgttatctgatttattacccatgaggatgttaccaagtattatatttacc-3´ and 5´-ggtaaatataatacttggtaacatcctcatgggtaataaatcagataacag-3´. The bacmids used to transfect the Sf9 insect cells (from *Spodoptera frugiperda*) were constructed in *Eschericia coli* DH10Bac cells.

Sf9 cells were cultured in suspension in HyQ SFX-Insect MP medium (Thermo Fisher Scientific, Waltham, MA) supplemented with 5% fetal bovine serum. Cells were infected with recombinant baculovirus-DNA and harvested and washed with phosphate buffered saline (PBS) approximately 60 hours after infection. Membranes were prepared from the cells using a modification of the Chu et al. [30] method as described in detail in [29]. To form the vesicles, the suspension was passed 10 times through a LIPEX™ Extruder (Northern Lipids Inc., Burnaby, BC, Canada) or 20 times through a 27 gauge needle. Protein concentration was measured using the Bio-Rad protein assay (Bio-Rad Laboratories Inc., Hercules, CA). Vesicles were stored at -75°C at protein concentrations of 3.5–5 mg/ml.

To improve the dynamic range of the assay, BCRP membranes were loaded with cholesterol similarly to Telbisz et al. [31], by incubating the membranes on ice for 20 minutes in the presence of the water-soluble cholesterol complex at a total concentration of 2.5 mM cholesterol. Excess of the complex was removed by diluting the mixture and centrifugation at 100 000 g at + 4°C for 1 h 15 min. The remaining membrane pellet was resuspended in membrane buffer (50 mM Tris–HCl pH 7.0, 50 mM mannitol, 2 mM EGTA). The resulting cholesterol concentration in the vesicles was roughly 100 μg/mg total protein as measured using the Amplex Red Cholesterol kit (Life Technologies, Carlsbad, CA).

### Substrate binding to BSA

The fraction of unbound substrate in the presence of BSA was studied with the rapid equilibrium dialysis (RED) device (Thermo Scientific, Waltham, MA). In the RED system, two chambers in a well are separated with dialysis membrane. The transporter substrate (CDCF, E_2_17βG or LY) was placed in the donor chamber in assay buffer with control membrane vesicles in the presence or absence of BSA to mimic the VT assay conditions. The final concentrations of BSA was 0.1% or 1.0%. Plain assay buffer was pipetted into the receiver chambers of all wells until the liquid level of both chambers was equal. For solubility reasons, the final composition of assay buffer included dimethyl sulfoxide (DMSO) up to 1.5% depending on substrate. Samples were incubated at 37°C on an orbital shaker. After 7 hour incubation, a small aliquot was collected from each receiver chamber for analysis. The relative fraction of unbound substrate (f_u_) was calculated as the ratio of concentrations in the receiver chambers from the samples with and without BSA.

### Vesicular transport (VT) assay

The effect of BSA was studied with three different substrates; 5(6)-carboxy-2,7-dichlorofluorescein (CDCF), and estradiol-17-β-D-glucuronide (E_2_17βG) were used with MRP2 and Lucifer yellow (LY) with BCRP. The assay was carried out as a modification of the PREDIVEZ™ Vesicular Transport Kit protocol (SOLVO Biotechnology, Szeged, Hungary). In short, vesicle stocks were thawed and diluted with assay buffer (40 mM MOPS–Tris pH 7.0, 60 mM KCl, 6 mM MgCl_2_). The substrate at various concentrations was added subsequently with 0.1% or 1.0% BSA (w/v), or an equivalent volume of deionized water for the control. 1.9 mM glutathione (GSH) was also included in the MRP2 assays. The well plate was preincubated at 37°C for 5–10 minutes after which half of the samples were treated with prewarmed plain assay buffer (non-ATP control), and the other half with prewarmed Mg-ATP-solution (final concentration 4 mM). Samples were subsequently incubated at 37°C for 8 minutes (E_2_17βG), 10 minutes (LY) and 30 minutes (CDCF) to coincide with the linear phase of uptake. Transport was terminated with ice cold washing buffer (40 mM MOPS-Tris pH 7.0 and 70 mM KCl). Samples were quickly transferred to the MultiScreenHTS-FB Plate Glass fiber 1.0 μm / 0.65 μm Durapore filter plate (Millipore, Molsheim, France). Wells were immediately washed five times with 200 μl washing buffer and dried. 0.1 M sodium hydroxide was used to elute LY and CDCF, which were detected by fluorescence measurement. E_2_17βG and was eluted with 0.1 M ammonium hydroxide and analyzed with mass spectrometry.

The modulation of MRP2 and BCRP uptake by fatty acid oleic acid was also studied with the VT assay. The assay was performed as above, but oleic acid was present at various concentrations. The probe concentration in these studies was 5 μM for CDCF and 50 μM for LY. Plain vehicle (70% ethanol) was used as a control.

### Membrane effects of oleic acid

Disruption of the membrane vesicles by oleic acid during the assay was evaluated using a modification of the VT assay. Vesicles were incubated with the substrates in the presence of ATP to load the vesicles. ATP-dependent transport was stopped using 1.2 mM sodium orthovanadate. Oleic acid or vehicle was added and after incubation for 10 min (LY) or 30 min (CDCF) the samples were diluted with washing buffer, filtered and washed as usual. The incubation time after stopping of the reaction was set as the same time that vesicles are incubated with oleic acid in the corresponding inhibition assays for MRP2 and BCRP.

### Analytical methods

CDCF and LY samples were measured with fluorometric detection using Varioskan Flash (Thermo Scientific, Vantaa, Finland). The excitation and emission wavelengths used for CDCF detection were 510 and 535 nm respectively. LY samples in 0.1 M sodium hydroxide were treated with an equal volume of 0.1 M hydrochloric acid before fluorometric analysis and detected with excitation at 430 nm and emission at 538 nm.

E_2_17βG samples for the BSA binding studies were analyzed by ultra-performance liquid chromatography (Acquity UPLC, Waters, Milford, MA). Acquity UPLC BEH Shield RP18 (130Å, 1.7 μm, 2.1 mm X 50 mm, Waters, Milford, MA) was used for E_2_17βG separation. Acetonitrile and phosphate buffers were used as mobile phase and flow rate was 0.5 ml/min. Samples were detected using UV/Vis spectroscopy using a wavelength of 218 nm for E_2_17βG.

Quantitative analyses of E_2_17βG vesicular transport assay samples were executed by ultra performance liquid chromatography-tandem mass spectrometry (UPLC-MS/MS) using a Waters ACQUITY UPLC I Class (Waters, Milford, MA) combined with a Waters XevoTM TQ-S MS triple quadrupole (Waters, Milford, MA). The UPLC system was equipped with a sample manager (maintained at 10°C), a binary solvent manager and a column thermostat (maintained at 26°C). Separations were performed on a 2.1 × 100 mm column, which was packed with 1.8 μm particles (ACQUITY UPLC HSS T3 C18, Waters, Milford, MA). The flow rate was 0.4 ml/min and the injection volume was 10 μl. Water with 0.1% formic acid (A) and ACN with 0.1% formic acid (B) were used as the mobile phases for the gradient elution. The gradient was as follows: from 0 to 3 min 5–100% B and from 3 to 7 min 100% B. Each run was followed by a 2 min re-equilibration period under initial conditions (5% B).

Mass spectrometric measurements were carried out using ESI source in negative ion mode. Capillary voltage was 1.5 kV, cone voltage 30 V. Desolvation temperature was set to 600°C, while desolvation gas flow was 600 l/hr and cone gas flow 150 l/hr. To reach enough sensitivity dwell time was set to 80 ms for the analyte and internal standards. Scan type was multiple reaction monitoring (MRM) and monitored ions were 447.1 > 74.8 (CE 25 eV) and 447.1 > 271.1 (CE 30 eV) for E_2_17βG and 348.9 > 268.9 (CE 35 eV) for estrone sulfate and 205.2 > 161.2 (CE 5 eV) for ibuprofen, which were used as internal standards.

### Data analysis

ATP-dependent transport was calculated as the difference between uptake in the presence and absence of ATP. All assays were performed in triplicates on a 96-well plate. Curve fitting to the data was performed with GraphPad Prism 6.04 (GraphPad Software Inc., La Jolla, CA). Substrate data was fitted with the Michaelis-Menten equation

$$v=\frac{V_{max}[S]}{K_{m}+[S]}$$ (1)

where v is the velocity of ATP-dependent transport, V_max_ is the maximal transport velocity, [S] is the substrate concentration and K_m_ is the Michaelis-Menten constant. In the case of E_2_17βG, which follows sigmoidal kinetics with MRP2 [32, 33], the equation was modified with h for Hill slope.

$$v=\frac{V_{max}{\left[S\right]}^{h}}{{K_{m}}^{h}+{\left[S\right]}^{h}}$$ (2)

Results from the kinetic studies are presented as relative transport values (%), which are calculated by normalizing all data points to the fitted V_max_ value of the control (0% BSA) and shown with standard deviation (SD). The statistical significance in the difference between V_max_ and K_m_ values calculated in the presence and absence of BSA was analyzed in GraphPad Prism using the extra-sum-of-squares F-test. The statistical significance in the difference between the uptake rate of 0.1% and 1.0% BSA treated vesicles was analyzed in GraphPad Prism using the unpaired t-test.

The oleic acid concentration required for 50% inhibition (IC_50_), was calculated from the concentration-response data using four parameter logistic regression,

$$Response={Response}_{min}+\frac{{Response}_{max}-{Response}_{min}}{1+{\left(\frac{\left[I\right]}{{IC}_{50}}\right)}^{h}}$$ (3)

where response is the control normalized ATP-dependent uptake (%), Response_min_ and Response_max_ represent the plateaus corresponding to minimal and maximal normalized uptake (%), [I] is the concentration of inhibitor (μM), IC_50_ is the inhibitor concentration required for a response half-way between minimal and maximal response (μM) and h is the Hill coefficient. The Response_min_ term was constrained to be larger than zero, where zero indicates full abolishment of uptake.

## Results

### Substrate binding to BSA

BSA binds a broad range of compounds and may thus decrease the concentration of free substrate in solution. In order to correct for the decrease in unbound concentration of the substrate in the assay, we measured the binding to BSA in assay conditions. The substrates used in this study showed none or low binding to 0.1% BSA at the concentrations studied (Table 1). CDCF was the only substrate to show a concentration dependent decrease in unbound concentrations in the concentration range studied. At 1.0% BSA, the decrease in unbound concentrations of CDCF was more pronounced, but LY remained completely unbound regardless of higher amount of BSA (Table 2). This binding of CDCF to 0.1% BSA was taken into account by correcting all concentration values with the average f_u_ of 0.94. The CDCF binding to 1.0% BSA was adjusted separately for each concentration. The unbound concentrations for the other compounds were not adjusted since the f_u_ was close to 1.0 and no trend for concentration dependency could be seen.

**Table 1:** Substrate binding to 0.1% BSA as measured using equilibrium dialysis. The f_u_ is calculated as the ratio of binding in the presence and absence of 0.1% BSA and is shown with standard deviation (SD) (n = 3).

Substrate	Concentration (μM)	f_u_ (± SD)
**5(6)-Carboxy-2,7-dichlorofluorescein (CDCF)**	1	0.92 (± 0.02)
	5	0.94 (± 0.02)
	50	0.96 (± 0.01)
**Estradiol-17-β-D-glucuronide (E**_**2**_**17βG)**	15	0.96 (± 0.04)
	50	1.00 (± 0.03)
	150	0.98 (± 0.04)
**Lucifer Yellow (LY)**	20	0.98 (± 0.01)
	200	0.98 (± 0.03)

**Table 2:** Substrate binding to 1.0% BSA as measured using equilibrium dialysis. The f_u_ is calculated as the ratio of binding in the presence and absence of BSA and is shown with standard deviation (SD) (n = 3).

Substrate	Concentration (μM)	f_u_ (± SD)
**5(6)-Carboxy-2,7-dichlorofluorescein (CDCF)**	5	0.43 (± 0.02)
	50	0.53 (± 0.02)
	200	0.73 (± 0.03)
**Lucifer Yellow (LY)**	5	1.03 (± 0.02)
	50	1.01 (± 0.03)
	200	1.03 (± 0.02)

### The effect of BSA in the VT assay

**Fig 1:**
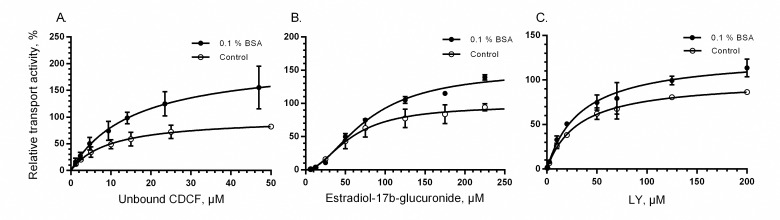
The effect of 0.1% BSA on uptake kinetics. Uptake of (A) 5(6)-carboxy-2’,7’-dichlorofluorescein (CDCF) in MRP2 vesicles, (B) Estradiol-17-β-glucuronide (E_2_17βG) in MRP2 vesicles and (C) Lucifer Yellow (LY) in BCRP vesicles in the absence (control, open circles) and presence of 0.1% BSA (closed circles). All assays were performed in triplicate and each point represents the mean (± SD) of 2–4 separate experiments normalized to the calculated V_max_ of the control. Curves represent the results from model fitting (see Materials and Methods).

The effect of BSA on MRP2 transport was examined with two commonly used MRP2 substrates, CDCF and E_2_17βG [34, 35]. A significant change in the ATP-dependent uptake of the substrate was seen for both substrates in the presence of 0.1% BSA (Fig 1). BSA increased V_max_ and K_m_ of CDCF by roughly 110% and 63%, respectively, but the difference was statistically significant only for V_max_ (Table 3). The observed effect was reproducible with different MRP2 vesicle batches.

**Table 3:** Kinetic parameters of substrate uptake into vesicles in the presence and absence of 0.1% bovine serum albumin (BSA). Parameters were calculated based on data from 2–4 separate experiments with GraphPad Prism and are reported as the best fit value (± standard error).

Transporter—substrate	K_m_ (μM)		V_max_ in the presence of 0.1% BSA (%) ^*a*^
	without BSA	with 0.1% BSA	
**MRP2 –CDCF**	9.6 (± 1.6)	15.6 (± 3.6)	210.4 (± 21.1) **
**MRP2 –E**_**2**_**17Βg**	56.7 (± 8.2)	77.2 (± 7.4)	154.7 (± 9.9) *
**BCRP–LY**	31.2 (± 3.2)	33.5 (± 5.1)	127.5 (± 6.2)**

^*a*^ The calculated V_max_ in the absence of BSA was set at 100%. For details see Materials and Methods

* p < 0.01 and

** p < 0.001 compared to the V_max_ in the absence of BSA

The uptake of endogenous estradiol metabolite, E_2_17βG, is known to exhibit sigmoidal kinetics in vesicles suggesting two binding sites on MRP2 [32, 33]. The sigmoidal kinetics was maintained after addition of BSA, but both the V_max_ and K_m_ increased, 55% and 36%, respectively (Table 3). Both K_m_ values were lower than the previously reported E_2_17βG K_m_ that range from 94 to 150 μM [33, 35–37].

**Fig 2:**
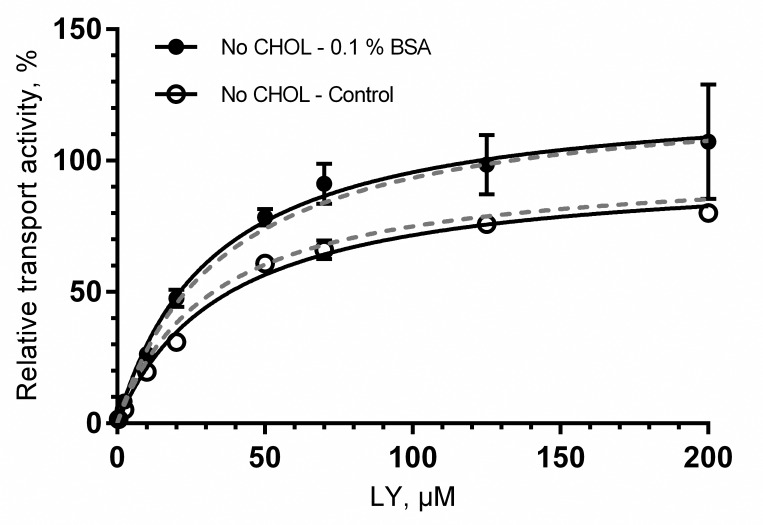
The effect of 0.1% bovine serum albumin (BSA) in BCRP vesicles not loaded with cholesterol. Uptake of Lucifer Yellow (LY) into BCRP vesicles not loaded with cholesterol is shown in the absence (control, open circles) and presence (closed circles) of 0.1% BSA. The V_max_ of control was set to 100% and the results normalized to this. Each point represents the mean ± SD, n = 3. Black solid curves represent the fitting of the data for vesicles without cholesterol loading and grey dashed curves represent the corresponding fitting from cholesterol loaded vesicles.

We selected Lucifer Yellow as a probe substrate to study BSA effects on BCRP transport as it is easily detected and used in commercial vesicular transport assay kits (SOLVO Biotechnology, Szeged, Hungary and GenoMembrane, Kanagawa, Japan). The presence of 0.1% BSA produced a small, but statistically significant increase of 28% in maximal LY uptake by BCRP (Fig 1, Table 3). As with MRP2, there was no significant change in K_m_ values. Since cholesterol is known to potentiate BCRP-mediated transport [38], we used BCRP vesicles loaded with cholesterol to observe the effect of BSA on highly active BCRP. However, no significant difference was detected in the effect of 0.1% BSA on LY uptake in the assays between cholesterol loaded and non-treated BCRP vesicles (Fig 2).

**Fig 3:**
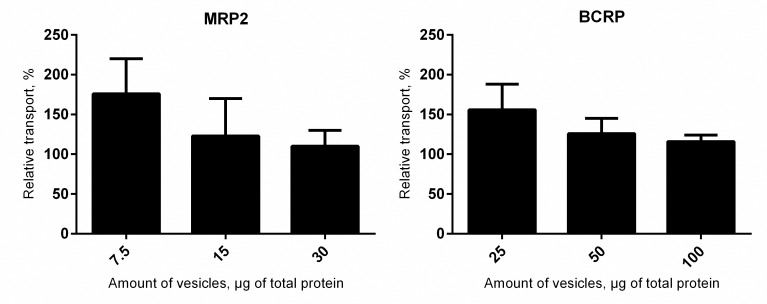
Relationship between the albumin effect and amount of vesicles used in the assay. Transport activity with varying amount of vesicles (measured as total protein in μg per well) were examined in the presence and absence of 0.1% BSA. Substrate concentrations in the assay were (left panel) 50 μM for CDCF (MRP2 vesicles) and (right panel) 200 μM for Lucifer Yellow (BCRP vesicles). Data is expressed as relative transport, normalized to the ATP-dependent uptake in the absence of BSA. Each bar represents the mean (± SD), n = 3–6.

Our in-house vesicle transport assays have been optimized to use lower protein amounts in the MRP2 assay than in the BCRP assay. In order to examine the sensitivity of the BSA effect to protein concentration, we tested the MRP2 and BCRP vesicles using three different protein amounts. For both transporters, the magnitude of BSA effect decreased as the protein amount increased (Fig 3).

**Fig 4:**
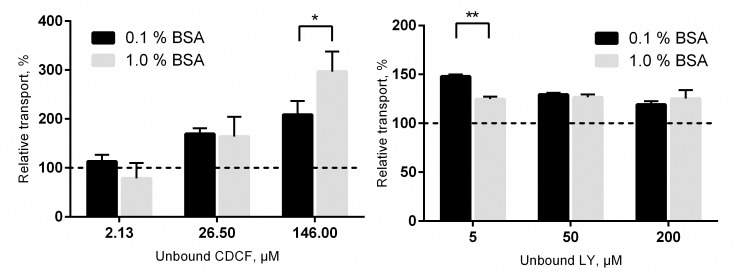
The comparison of 0.1% and 1.0% BSA effect on uptake kinetics. (Left panel) CDCF uptake into MRP2 vesicles, (right panel) Lucifer Yellow (LY) uptake into BCRP vesicles. Each bar represents the mean (± SD) of 2–3 separate experiments. The uptake rate of the control (0% BSA) was set to 100% and uptake rates in the presence of 0.1% and 1.0% BSA were normalized to control. * p < 0.05 and ** p < 0.01 compared to the uptake in the absence of BSA.

To determine whether the albumin effect is dependent on the BSA concentration, we compared CDCF and LY uptake in presence of 0.1 and 1.0% BSA. The higher BSA concentration resulted in a larger uptake only at the highest tested CDCF concentration (Fig 4, left panel). In contrast, at the lowest LY concentration, 0.1% BSA had a larger effect on the transport than 1% BSA.

### Inhibition of transport by oleic acid

The exact mechanism of the albumin effect on metabolic enzymes like UGTs and CYPs is unknown, but as one key physiological role of albumin is the transport of fatty acids, it is proposed that albumin binds inhibitory free long chain fatty acids that are liberated from cell membranes during the production of membrane vesicles [27, 39–42]. For a similar mechanism of action to be applied in the case of ABC transporters, it would require that the fatty acids present in the vesicle preparation have an inhibitory potential on MRP2 and BCRP. In the case of Sf9 cells that we have used, the most abundant fatty acid is oleic acid, comprising 48% of the lipid composition [43].

**Fig 5:**
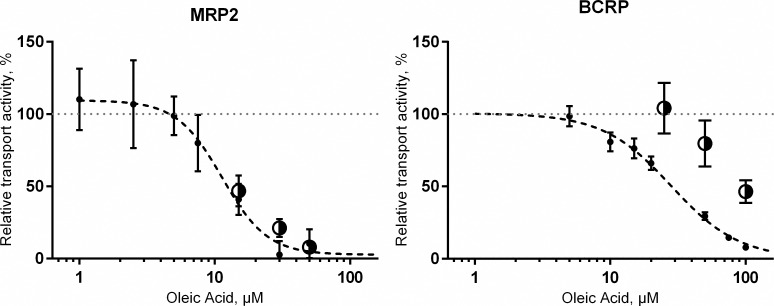
Inhibition of 5 μM CDCF uptake into MRP2 vesicles and 50 μM LY uptake into BCRP vesicles by oleic acid. Data is presented as a relative transport activity normalized to uptake in the absence of oleic acid. Small, closed circles show results from inhibition studies and larger black/white circles represent the retention of CDCF or LY in vesicles in studies on unspecific membrane effects of oleic acid (for details see Materials and Methods). All data points were assayed in triplicate and curve fitting was performed using the four parameter logistic equation. Graphs show the results from a representative experiment and data is represented as mean ± SD. IC_50_ (95% confidence interval) for BCRP was 28.2 μM (21.7–37.7). In the case of MRP2, the IC_50_ was not applicable.

Oleic acid decreased the uptake of substrates in both MRP2 and BCRP vesicles in a concentration dependent manner (Fig 5). However, since fatty acids can interfere with the membrane vesicles and exert their apparent inhibitory effect by disturbing the vesicle structure [44], we studied retention of the substrate in the vesicles after inhibition of the transporters with sodium orthovanadate and subsequent addition of oleic acid. In MRP2 vesicles, the CDCF retention after the addition of oleic acid followed the decrease in uptake observed in the inhibition assay (Fig 5, left panel). This suggests that the observed inhibitory effect of oleic acid on MRP2 is due to the breakage of vesicles. The cholesterol-loaded BCRP vesicles were less affected by oleic acid, even though at increasing oleic acid concentration, less LY was retained in the vesicles (Fig 5, right panel).

## Discussion

Our results show that the BSA effect for MRP2 and BCRP is displayed mainly through changes in V_max_. This is in contrast to the results obtained with the metabolic enzymes where a decrease in K_m_ is usually the most pronounced effect of albumin [27, 28, 39–42, 45, 46]. Instead, transporter-mediated uptake of both CDCF and LY showed a slight increase in K_m_ in the presence of BSA, although the change was not statistically significant. An increase in (unbound) K_m_ with albumin has earlier been noted for UGT1A1 3-glucuronidation of estradiol in human liver microsomes [47] and UGT1A4 N2-glucuronidation of lamotrigine in recombinant UGT1A4 expressed in HEK293 [28], but the reason for this deviating behavior is unknown.

The effect of BSA in this study was lower for MRP2 and BCRP (maximum 3-fold) than what has previously been reported for metabolic enzymes. Albumin caused a > 20-fold decrease in the K_m_ of 6-hydroxyindole metabolism by UGT2A1 [48] as well as 10- and 7-fold increase in affinity of zidovudine and lamotrigine to UGT2B7 [28, 45]. Manevski et al. [48] also reported an up to 7-fold increase in V_max_ of entacapone metabolism by UGT1A7. The effect of albumin that is observed on the efflux transporter activity is more comparable to the results obtained with CYPs, where albumin decreased the K_m_ of phenytoin and paclitaxel hydroxylation in human liver microsomes by 4- and 3-fold respectively [27, 41]. The smaller effect observed for the efflux transporters could result from the lower albumin concentration used here (1.0%), than in many of the metabolic studies that use higher albumin concentrations, up to 2%. At lower concentrations, like the 0.1 and 1.0% albumin concentration used in our studies, the albumin effect is often less pronounced for the metabolic enzymes than at higher albumin concentrations [39, 40, 46, 48]. However, in our work, the addition of 1.0% BSA did not increase substrate uptake except in the highest substrate concentration for MRP2. In contrast, the 0.1% BSA enhanced LY uptake more than 1.0% BSA, when the LY concentration was low (5μM).

BSA has a slightly larger effect with the studied MRP2 assay than the BCRP assay, but that might be due to the different protein amounts used in the assays. In our assays, the total amount of protein in the MRP2 and BCRP vesicles were 7.5 μg and 50 μg, respectively. However, the direct comparison of vesicle amounts between MRP2 and BCRP would not be feasible since the vesicle preparations are not the same. Moreover, we investigated if the cholesterol treatment could cause the observed differences. Apart from changing the properties of the membrane, cholesterol addition may remove some of the free fatty acids or other lipophilic compounds in the vesicle preparation through binding to the cyclodextrin complex used to solubilize cholesterol during cholesterol loading. However, as the cholesterol loading does not appear to influence the observed BSA effect in BCRP vesicles, it is unlikely that it would cause the difference between BCRP and MRP2 either. It is possible that the difference in the BSA effect on the MRP2 and BCRP assays are due to substrate- or transporter-dependency, as has been observed for UGTs [48].

Albumin is proposed to influence the activity of metabolic enzymes through binding of inhibitory free fatty acids that originate from the vesicles. Oleic acid, the most abundant fatty acid in Sf9 cells, was able to inhibit BCRP transport activity, but our assay was unsuitable to investigate the effect of oleic acid on MRP2, since oleic acid interfered with the membrane integrity of MRP2 vesicles. The calculated IC_50_ value for BCRP, 28.2 μM, should be considered as an apparent value, because the concentration of oleic acid inherently present in the vesicle preparation is unknown and high concentrations of oleic acid interferes with the membrane structure of the cholesterol-loaded BCRP vesicles. High concentration (> 1 mM) of oleic acid was previously reported to decrease BCRP-mediated transport of mitoxantrone in Caco-2 cells [49] and rosuvastatin in sandwich-cultured hepatocytes [50], but we are unaware of previous reports on the interaction between oleic acid and MRP2. Based on our results, the free oleic acid present in the vesicle preparations could weakly inhibit BCRP and removal of free fatty acids by addition of BSA could lead to the effect of BSA observed on BCRP.

In summary, we have shown that the addition of BSA to the vesicular transport assay has an effect on the maximal uptake rate of MRP2 and BCRP substrates. Oleic acid inhibited BCRP, which is in line with the proposed mechanism behind the albumin effect on metabolic enzymes. The inhibitory effect of oleic acid on MRP2 could not be investigated, as oleic acid damaged the MRP2 vesicles. The effect of BSA on substrate uptake was dependent on the vesicle amount, determined by protein concentration, while increasing BSA concentration did not significantly improve substrate uptake. Even though albumin clearly has an effect in the vesicle transport assay, it may not have a very large effect on the estimated clearance of transporter substrates. Since both V_max_ and K_m_ values of the substrates increased in our study, the calculated transporter mediated clearance would be only slightly altered (30% higher) compared to conditions without BSA, considering linear kinetics where clearance is the ratio of V_max_ to K_m_. Such small alterations in clearance are unlikely to have a major role in *in vitro*—*in vivo* predictions of the MRP2- and BCRP -mediated transport, as they are easily lost in other variability such as inter-laboratory variation. However, as the albumin effect has been observed to be both transporter- and substrate-dependent for the metabolic enzymes, it may have a larger impact with other substrates and/or transporters and should therefore be kept in mind when BSA is included in the vesicular assay for example as a solubilizer.

## Abbreviations

BCRP: breast cancer resistance protein (ABCG2)
BSA: bovine serum albumin
CDCF: 5(6)-carboxy-2’,7’-dichlorofluorescein
E_2_17βG: estradiol-17-β-D-glucuronide
f_u_: fraction unbound
LY: Lucifer yellow
MRP2: multidrug resistance associated protein 2 (ABCC2)
VT assay: vesicular transport assay
